# Autophagy for Better or Worse during Infectious Diseases

**DOI:** 10.3389/fimmu.2013.00205

**Published:** 2013-07-19

**Authors:** Irina Caminschi, Christian Münz

**Affiliations:** ^1^Centre for Immunology, Burnet InstituteMelbourne, VIC, Australia; ^2^Institute of Experimental Immunology, University of ZürichZürich, Switzerland

Autophagy describes at least three metabolic pathways that deliver cytoplasmic constituents for lysosomal degradation (1). While micro- and chaperone-mediated autophagy engulf or translocate cytosolic material at the late endosomal or lyosomal membrane, respectively, macroautophagy can use different membrane sources, including endoplasmic reticulum, Golgi, plasma membrane, mitochondria, and outer nuclear membrane to enclose large portions of the cytoplasm in autophagosomes (2). These double membrane surrounded vesicles are generated *de novo* around macroautophagy cargo like damaged organelles, protein aggregates, and cytosolic pathogens, and more than 30 autophagy related (Atg) gene products are involved in their formation and fusion with lysosomes. The series of review articles in this *Frontiers in Immunology* e-book will high-light how regulation of macroautophagy during infections results in cytosolic restriction of pathogens, sometimes supports their replication and is connected to innate immune activation as well as adaptive immune responses to these environmental insults.

In the first set of reviews, the interactions of pathogens with macroautophagy will be discussed. Dengjel and coworkers will summarize the regulation of macroautophagy by influenza A virus and how this changes macroautophagic flux (3). This review particularly focuses on the sequential recruitment of substrates to autophagosomes and interference by influenza A virus. A second review by Biard-Piechaczyk and coauthors will then discuss the different functions that macroautophagy has during human immunodeficiency virus (HIV) infection of T cells and macrophages (4). Differences in the viral replication within these two host cells appear to determine the role that macroautophagy plays in HIV propagation in these targets. Furthermore, Faure and coworkers will high-light that there are certain nodes in the macroautophagy network that are targeted by many viruses (5). Particularly the GTPase IRGM will be discussed. Moreover, Taylor and colleague will discuss the regulation of macroautophagy by herpesviruses (6). Atg6/Beclin-1 targeting by these pathogens has resulted in fascinating insights and tools to dissect macroautophagy. Finally, this block of reviews is concluded with a text by Sasakawa and coworkers (7). They discuss the restriction of bacterial dissemination by macroautophagy and the counter responses of the bacteria aimed at escaping these immune measures. Thus, many pathogens regulate and are restricted by macroautophagy during infection.

A second set of reviews explores the role of macroautophagy in immune responses. Innate immune recognition, resulting cytokine production, antigen processing for MHC presentation, and autoimmunity will be discussed in this block. Lee and coworkers will discuss how macroautophagy regulates pathogen detection by the immune system (8). Both the turnover of cytosolic receptors of pathogen associated molecular patterns (PAMPs) and the transport of PAMPs to vesicular receptors is affected by macroautophagy. Moreover, early innate cytokine production is regulated by this pathway. Harris discusses the influence of macroautophagy on IL-1 production (9). Furthermore, Villadangos and colleague highlight the role of macroautophagy in innate and adaptive immunity, covering its role in antigen processing, as well as in T and B cell physiology (10). Expanding on some of these themes, Albert and co-worker summarize the evidence that macroautophagy contributes to exogenous antigen cross-presentation onto MHC class I molecules (11) and focus on the role of the antigen donor cell. In a second review on antigen processing via macroautophagy, the role of this pathway in MHC class II antigen processing will be discussed (12). Particularly, its contribution to both intracellular and extracellular antigen processing will be considered. Beyond antigen processing, He and colleagues will review the role for macroautophagy in lymphocyte development and function (13). This article focuses on the role of macroautophagy in T cells. Finally, Eissa and coauthor summarize how macroautophagy alterations might lead to hyperreactivity to gut commensals and autoimmunity (14). In this respect genetic predisposition to Crohn's disease, which affects essential autophagy genes, will be discussed. Thus, this second set of reviews captures the breadth of functions for macroautophagy in immunity.

Macroautophagy is, therefore, not only an essential metabolic pathway, but has also been used during the co-evolutionary struggle between pathogens and their hosts to benefit one or the other. One would predict that it may also play a role in many other infectious diseases, and consequently, could serve as a therapeutic target. However, since macroautophagy can serve the immune system or be exploited by the pathogen, its role has to be characterized for every single different pathogen in order to predict the effect its manipulation would have during infection.
